# Introduction of peripheral nitrogen atoms to cyclo-*meta*-phenylenes

**DOI:** 10.3762/bjoc.20.103

**Published:** 2024-05-24

**Authors:** Koki Ikemoto, Hiroyuki Isobe

**Affiliations:** 1 Department of Chemistry, The University of Tokyo, Hongo 7-3-1, Bunkyo-ku, Tokyo 113-0033, Japan

**Keywords:** cross coupling, macrocycles, nitrogen doping, UV–vis spectroscopy, X-ray charge density analysis

## Abstract

Cyclo-*meta*-phenylenes doped with nitrogen atoms at the periphery were designed and synthesized. The syntheses of the macrocyclic structures were achieved with one-pot Suzuki–Miyaura coupling to arrange phenylene rings and pyridinylene rings in an alternating fashion. Analyses with UV–vis spectroscopy showed changes in the photophysical properties with nitrogen doping, and X-ray crystallographic analyses experimentally revealed the presence of biased charges on the peripheral nitrogen atoms.

## Introduction

**Figure 1:**
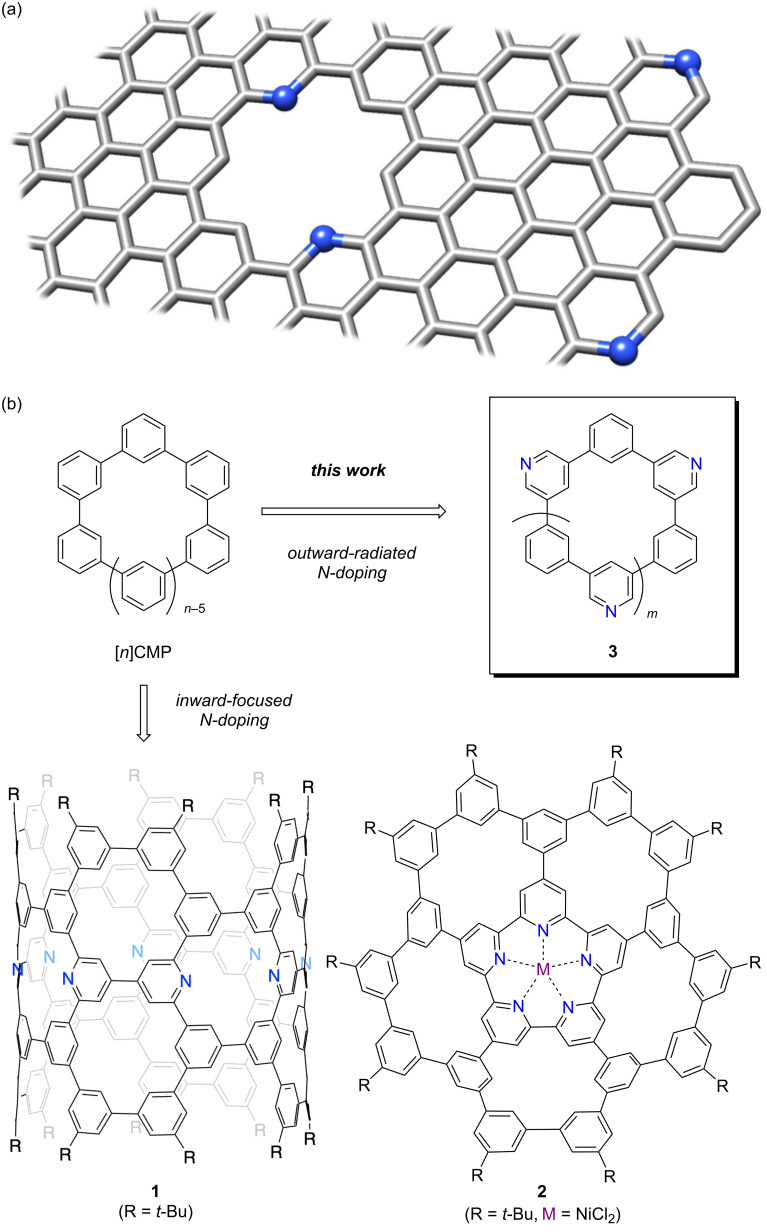
Nitrogen-doped nanocarbons. (a) Schematic illustration of pyridinic nitrogen atoms installed at the interior and periphery of a graphene sheet. (b) Phenine nanocarbon molecules with nitrogen dopants.

Graphitic carbonaceous sheets of graphene continue to attract considerable attention, which lead us to explore structural defects such as heteroatom doping and porous defects for unique properties and functions. For instance, with nitrogen atoms as dopants [1–3], a range of applications, such as electrocatalysis [4] and gas storage [5], has been exploited. Although the locations of nitrogen, in addition to the types, including pyridinic, pyrrolic and graphitic nitrogen, play important roles in determining the properties and functions (Figure 1a), top-down, physical production does not enable control of the doped structures with atomic precision. The bottom-up chemical syntheses of molecular nanocarbons have thus become attractive for controlling the nitrogen-doped structures embedded in large, molecular π-systems [6–7]. As a versatile synthetic strategy for defective molecular nanocarbons, we recently introduced phenine design [8–9], which allowed us to introduce nitrogen dopants, as was demonstrated with nitrogen-doped phenine nanocarbons such as **1** and **2** [10–11]. The nitrogen dopants were introduced in an inward-focused manner to decorate the inner rims of [*n*]cyclo-*meta*-phenylenes ([*n*]CMP) (Figure 1b) and captured other entities such as protons and metal atoms at the porous defect. In this study, the nitrogen dopants were installed in an outward-radiated manner, which expands the structural diversity to exploit the chemistry at the periphery of [*n*]CMP (Figure 1b). Through the use of Suzuki–Miyaura coupling for macrocyclisation, pyridinyl and phenylene rings were assembled in an alternating fashion, which afforded nitrogen-doped [*n*]CMPs (**3**) containing outward-radiating nitrogen dopants. The properties and structures were investigated with UV–vis spectroscopy and X-ray crystallography, which revealed the fundamental properties of the nitrogen dopants in the macrocyclic structures.

## Results and Discussion

**Scheme 1:**
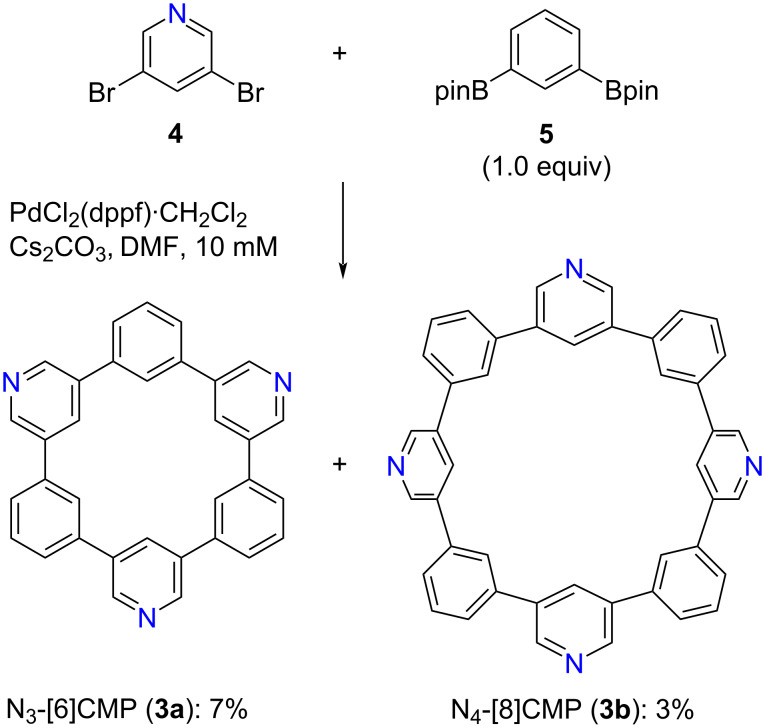
Syntheses of **3a** and **3b**.

Nitrogen-doped [*n*]CMPs, **3a** and **3b**, were synthesized via one-pot Suzuki–Miyaura coupling [12] (Scheme 1). Previously, we synthesized [*n*]CMPs with inward-focused nitrogen dopants by using Suzuki–Miyaura coupling with Pd(PPh_3_)_4_ as the catalyst [13] and applied this method to outward-radiated congeners in this work. However, a MALDI-TOF MS analysis of the crude mixture showed that macrocyclisation did not complete to afford a complex mixture containing noncyclic, linear oligomers (Figure S1, Supporting Information File 1). After examining the Pd-catalysts, we found that macrocyclisation with PdCl_2_(dppf)·CH_2_Cl_2_ worked best and afforded cyclic congeners from N_3_-[6]CMP to N_6_-[12]CMP (Figure S1, Supporting Information File 1) and isolated N_3_-[6]CMP (**3a**) and N_4_-[8]CMP (**3b**) in 7% and 3% yields, respectively [14].

**Figure 2:**
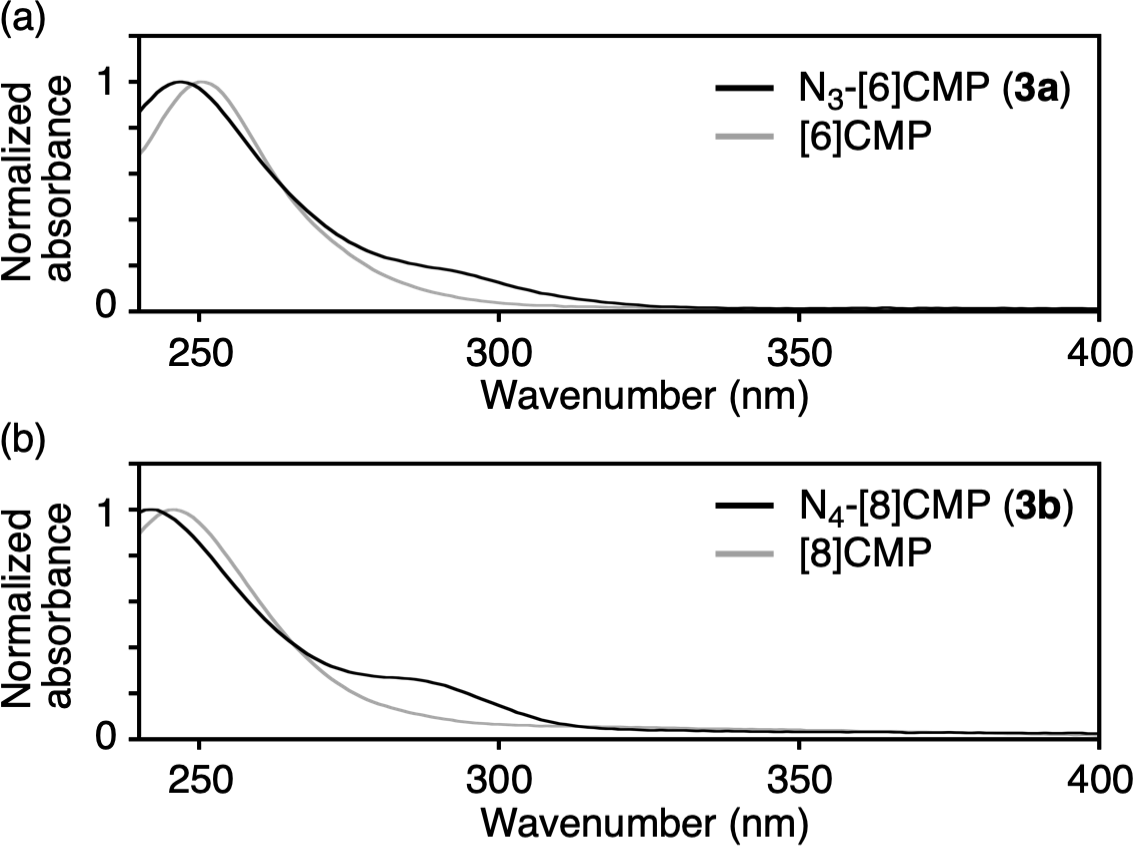
Photophysical properties of **3a** and **3b**. (a) UV–vis spectrum of **3a** in CHCl_3_. (b) UV–vis spectrum of **3b** in CHCl_3_. For reference, the spectra of [6]CMP and [8]CMP from the literature are also shown in gray [15].

Comparisons of the UV–vis spectra of the doped and undoped congeners revealed dopant-induced changes in the electronic properties. The UV–vis spectra of **3a** and **3b** were recorded in chloroform and are shown in Figure 2, with spectra of the undoped [*n*]CMP congeners shown as references [15]. The nitrogen-doped [*n*]CMPs **3a** and **3b** commonly showed minor yet new absorptions at approximately 280 nm, with major absorptions appearing at 250 nm (Figure 2). As shown with the reference spectra of [6]CMP and [8]CMP, the absorption at 280 nm was absent for the corresponding hydrocarbon congeners. These results showed that the nitrogen-dopants induced novel transitions for photoexcitation.

**Figure 3:**
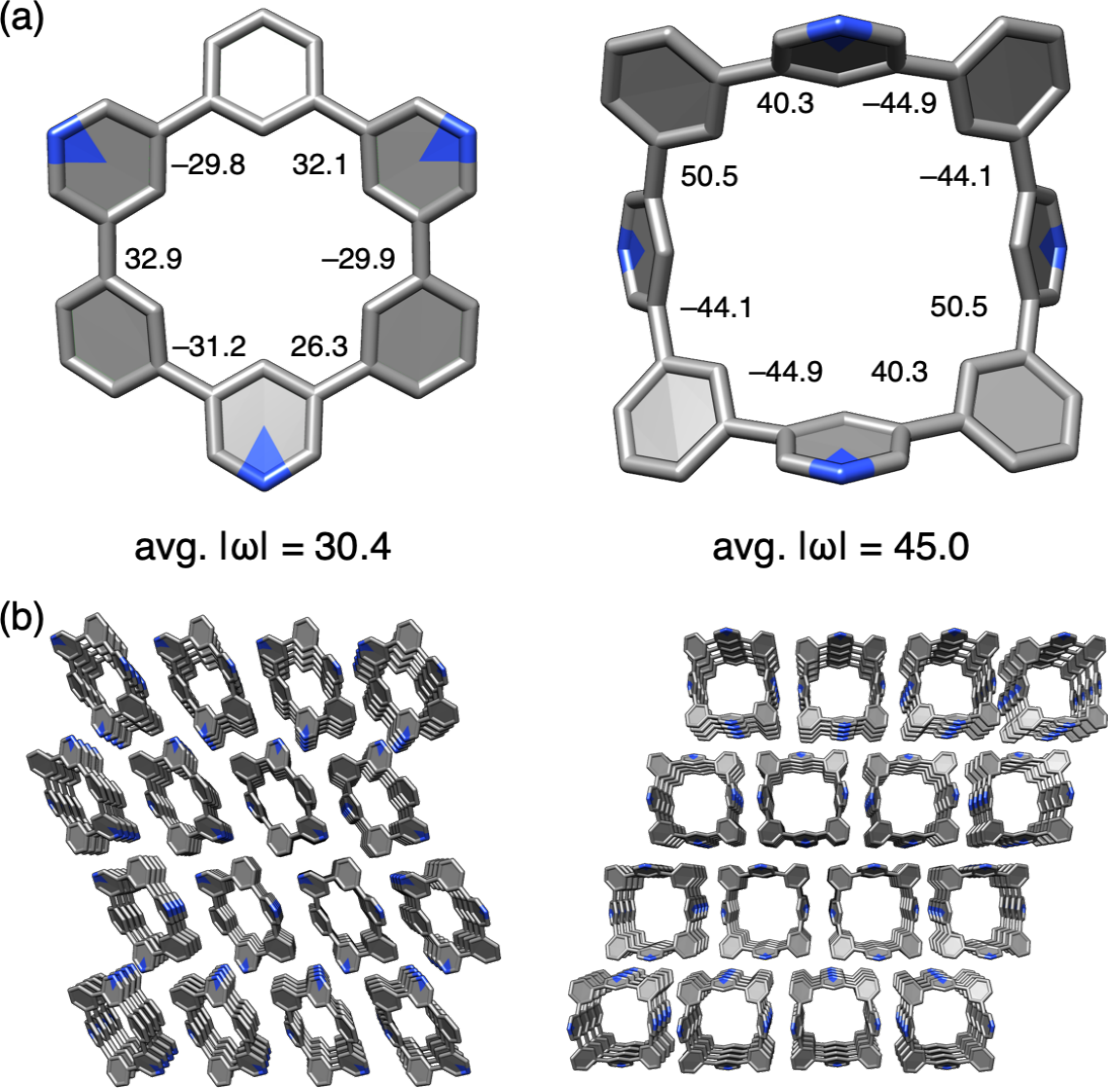
Crystal structures of **3a** and **3b**. (a) Molecular structures. Biaryl dihedral angles (ω) are shown. (b) Packing structures. Chloroform molecules in the crystal of **3b** are omitted for clarity.

Crystallographic analyses revealed the structural features of nitrogen-doped [*n*]CMPs. The crystal molecular structures of **3a** and **3b** are shown in Figure 3. The hexagonal macrocyclic structure of **3a** showed a chair-like conformation with alternating biaryl dihedral angles showing +/– values. The octagonal structure of **3b** exhibited a saddle-like conformation with an average dihedral angle of 45.0°, which was slightly larger than that of **3a** (30.4°). The shapes of nitrogen-doped [*n*]CMPs did not deviate from those of hydrocarbon [*n*]CMPs, with similar average dihedral angles (32.4° for [6]CMP and 40.6° for [8]CMP) [15]. Likewise, the crystal packings of **3a** and **3b** resembled those of the hydrocarbon congeners, forming one-dimensional columns of stacked macrocycles (Figure 3b).

**Figure 4:**
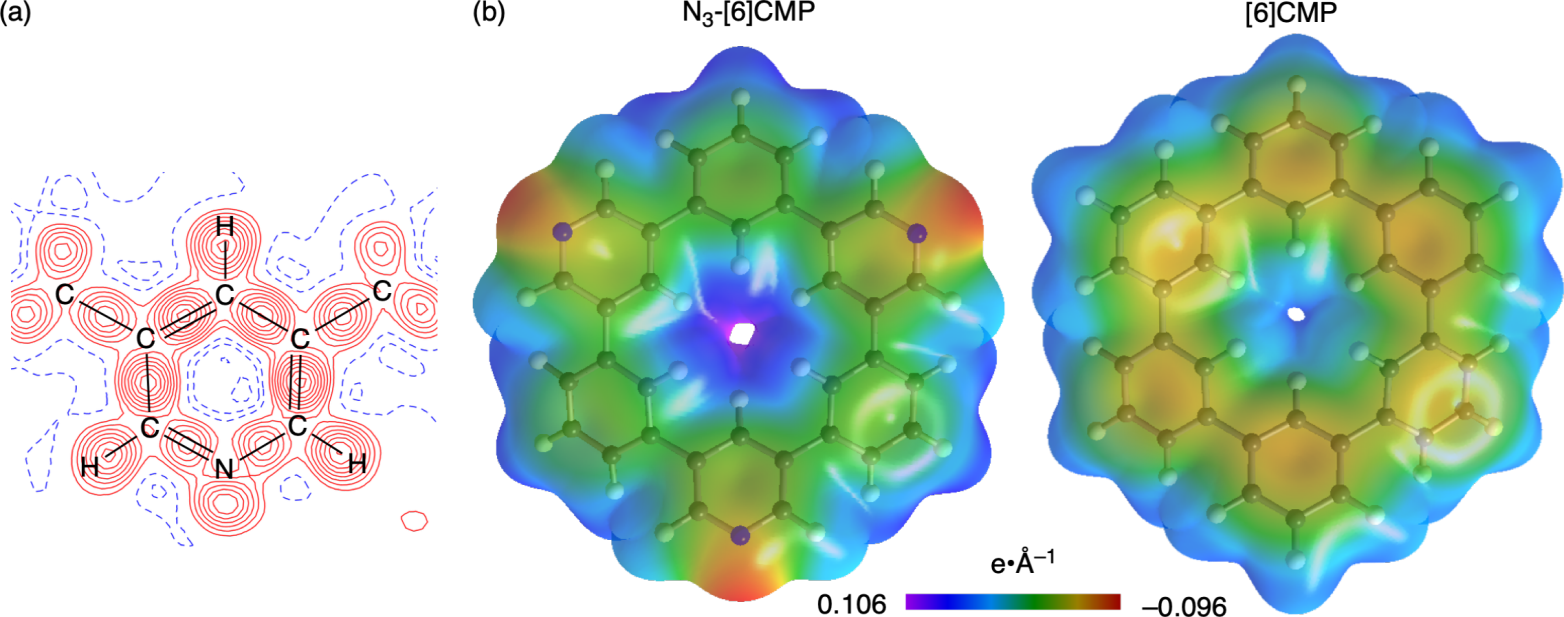
X-ray charge density analyses of **3a** and [6]CMP. (a) Deformation map (*F*_o_ – *F*_c_) of a pyridine ring in **3a** (contour interval: 0.05 e·Å^−3^, positive: red, negative: blue). (b) Electrostatic potential maps mapped on the 0.0067 e·Å^−3^ isosurface for the electron density.

The electronic effects of the nitrogen dopants in **3a** were examined with X-ray charge density analyses [10,16]. For the crystal structures shown in Figure 3, we used a standard method with spherical independent atom models (IAM) [17], whereas for the charge density analyses, we used the transferrable aspherical atom models (TAAM) from the Hansen and Coppens formalism [18–19]. The TAAM analysis with parameters from the University at Buffalo pseudoatom databank (UBDB) [20] was performed on XD2016 [21] to obtain an *R* factor of *R*(*F*) = 0.0269, which was better than that of the IAM on XD2016 with *R*(*F*) = 0.0382. As shown in Figure 4, the TAAM analysis allowed us to obtain a deformation map that located bonding and lone-pair electron densities of the nitrogen atom (Figure 4a). The analyses also allowed us to visualise experimental electrostatic potential (ESP) maps to reveal the presence of negative potentials on the nitrogen atoms (Figure 4b). For comparison, we performed TAAM analyses of hydrocarbon [6]CMP by reanalysing previous diffraction data [15] and obtained the corresponding ESP maps. A comparison of the ESP maps of **3a** and hydrocarbon [6]CMP showed induction of biased densities by the nitrogen dopants. Similar biased densities were previously found to be critical in determining the packing structures of nitrogen-doped π-systems to make parallel-displaced configurations preferred over T-shaped stackings [22]. In our study, we observed that pyridine–pyridine stacks were preferred in the crystal stacking (Figure 3), which might be attributed to the biased ESPs on the macrocycles.

**Figure 5:**
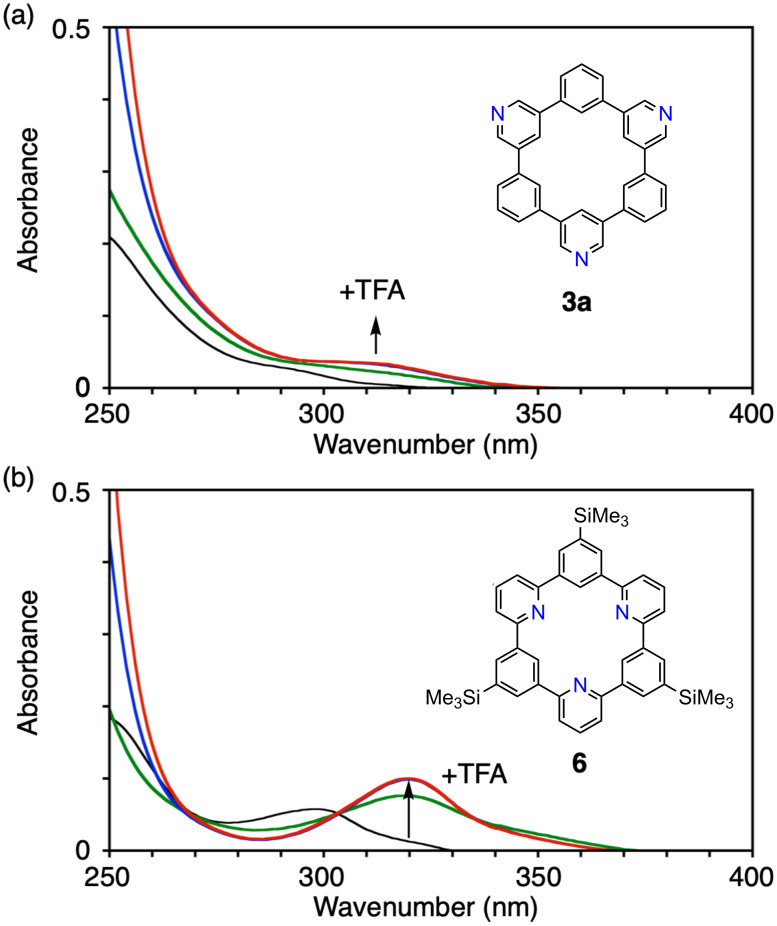
Response towards acid treatment with nitrogen-doped CMPs. (a) Absorption spectra of **3a** (CHCl_3_, 2.3 × 10^−6^ M) in the absence (black) and presence of trifluoroacetic acid (green: 4.3 × 10^−2^ M, blue: 2.1 × 10^−1^ M, red: 4.2 × 10^−1^ M). (b) Absorption spectra of **6** (CHCl_3_, 1.9 × 10^−6^ M) in the absence (black) and presence of trifluoroacetic acid (green: 4.3 × 10^−2^ M, blue: 2.1 × 10^−1^ M, red: 4.2 × 10^−1^ M).

Finally, we found that nitrogen locations altered chemical characteristics of nitrogen-doped CMPs. Thus, when trifluoroacetic acid (TFA) was added to a solution of **3a** in chloroform, bathochromic shifts in UV spectra were observed, indicating protonation-induced changes in the electronic properties [10]. Because of the weakly acidic nature of pyridinic nitrogen atoms, an excess amount of acid was necessary for this effect to be observed with a maximum equivalent of TFA at 2 × 10^5^, and the absorption band at the longest wavelength gradually shifted as shown in Figure 5a. When we added TFA to a solution of a reference compound **6** having inward-focused nitrogen atoms with a maximum equivalent of TFA at 2 × 10^5^ [13], bathochromic shifts were also observed. However, unlike the case with **3a**, gradual absorption shifts were not observed, and the absorption changed from 299 nm to 320 nm with an isosbestic point at 303 nm as shown in Figure 5b. This observation indicated that the protonation-induced change of UV spectra for **6** involved two equilibrating states, which most likely originated from single protonation at the centre of the CMP pore. On the other hand, the gradual absorption shifts observed with **3a** might thus be ascribed to the presence of multiple protonated species involved in the equilibrium. These results show that the coordination chemistry associated with nitrogen dopants may well be controlled by the locations and directions of the nitrogen atoms.

## Conclusion

Macrocycles with nitrogen atoms doped at the periphery were designed and synthesised, and their electronic properties were experimentally investigated with UV–vis and X-ray charge density analyses. The changes in the UV–vis transitions caused by nitrogen dopants may be useful for designing doped materials for optical applications, and the biased ESPs on the macrocycles should also be considered for material design. Nitrogen-induced π-stacking may also enable exploration of molecular assemblies. The experimental lone-pair electron densities were directed outward in **3a** and could be used as linkers for metal atoms to assemble trigonal pyramidal macrocycles, for instance, in networks of metal organic frameworks [23–24]. Investigations of the nitrogen dopants in molecular nanocarbons should enrich the chemistry of nanocarbons.

## Experimental

Syntheses of N_3_-[6]CMP (**3a**) and N_4_-[8]CMP (**3b**) [14]: A mixture of 3,5-dibromopyridine (**4**, 7.11 g, 30.0 mmol), diborylbenzene (**5**, 9.90 g, 30.0 mmol), PdCl_2_(dppf)·CH_2_Cl_2_ (2.50 g, 3.0 mmol), and Cs_2_CO_3_ (48.9 g, 150 mmol) in 3.0 L of DMF was stirred at 110 °C for 24 h. After the addition of H_2_O (2.5 L) and CHCl_3_ (3.0 L), the precipitate was removed by filtration. The organic layer was separated, dried over Na_2_SO_4_, and concentrated in vacuo. To eliminate the soluble by-products, the crude material was first washed with CHCl_3_ (100 mL), and a residue comprising **3a** and **3b** was obtained. The residue was then suspended in CHCl_3_ (100 mL) and sonicated for 10 min. After separating the solid and the filtrate, each sample was purified as follows: The former was subjected to Soxhlet extraction with CHCl_3_ overnight to give **3a** in 6% yield (272 mg, 0.592 mmol) after the extraction. The latter was purified by silica gel short path and GPC (column: YMC-GPC T30000-40 + T4000-40 + T2000-40, eluent: CHCl_3_, flow rate: 30 mL/min) to give **3a** in 1% yield (52.8 mg, 0.115 mmol) and **3b** in 3% yield (118 mg, 0.193 mmol). In total, **3a** was obtained in 7% yield (325 mg, 0.707 mmol). N_3_-[6]CMP (**3a**): ^1^H NMR (CDCl_3_, 600 MHz) δ 9.05 (d, *J* = 2.1 Hz, 6H), 8.58 (t, *J* = 2.1 Hz, 3H), 8.23 (t, *J* = 2.1 Hz, 3H), 7.89 (dd, *J* = 7.6, 2.1 Hz, 6H), 7.69 (t, *J* = 7.6 Hz, 3H); ^13^C NMR (CDCl_3_, 150 MHz) δ 146.6 (CH), 138.3, 135.7, 133.9 (CH), 130.2 (CH), 127.5 (CH), 125.9 (CH); HRMS (APCI) (*m*/*z*): [M + H]^+^ calcd. for C_33_H_22_N_3_, 460.1808; found, 460.1808. N_4_-[8]CMP (**3b**): ^1^H NMR (CDCl_3_, 600 MHz) δ 8.81 (d, *J* = 1.4 Hz, 8H), 8.13 (t, *J* = 1.4 Hz, 4H), 7.76 (s, 4H), 7.66–7.73 (m, 12H); ^13^C NMR (CDCl_3_, 150 MHz) δ 147.9 (CH), 139.2, 137.0, 133.5 (CH), 129.9 (CH), 127.5 (CH), 127.3 (CH); HRMS (APCI) (*m*/*z*): [M + H]^+^ calcd. for C_44_H_29_N_4_, 613.2387; found, 613.2366.

## Supporting Information

File 1 Experimental and copies of spectra.

File 2 Crystallographic data of N_3_-[6]CMP (**3a**) analysed by SHELX (CCDC 2335441).

File 3 Crystallographic data of N_4_-[8]CMP (**3b**) analysed by SHELX (CCDC 2335442).

File 4 Crystallographic data of N_3_-[6]CMP (**3a**) analysed by XD2016 (CCDC 2335443).

File 5 Crystallographic data of [6]CMP analysed by XD2016 (CCDC 2335444).

## Data Availability

All data that supports the findings of this study is available in the published article and/or the supporting information to this article. The crystallographic data were deposited in the Cambridge Crystallographic Data Centre (CCDC 2335441–2335444). The data can be obtained free of charge from the CCDC via http://www.ccdc.cam.ac.uk/data_request/cif.
