# Supplementary material for: Introduction of peripheral nitrogen atoms to cyclo-meta-phenylenes
Source: Beilstein J Org Chem. 2024 May 24;20:1207–12. doi: 10.3762/bjoc.20.103 (PMC11181282; doi:10.3762/bjoc.20.103)
Supplement: File 1 — Experimental and copies of spectra. [file Beilstein_J_Org_Chem-20-1207-s001.pdf]

BEILSTEIN JOURNAL OF ORGANIC CHEMISTRY

Supporting Information

for

Introduction of peripheral nitrogen atoms to cyclo-*meta*-phenylenes

Koki Ikemoto and Hiroyuki Isobe

Beilstein J. Org. Chem. **2024,** *20,* 1207–1212. doi:10.3762/bjoc.20.103

Experimental and copies of spectra

License and Terms: This is a supporting information file under the terms of the Creative Commons Attribution License (https://creativecommons.org/licenses/by/4.0). Please note that the reuse, redistribution and reproduction in particular requires that the author(s) and source are credited and that individual graphics may be subject to special legal provisions.
The license is subject to the *Beilstein Journal of Organic Chemistry* terms and conditions: (https://www.beilstein-journals.org/bjoc/terms)

Table of contents

Supplementary data....S2

Methods....S4

Materials....S4

Crystallography....S4

Spectra....S10

Supplementary references....S14

Supplementary data

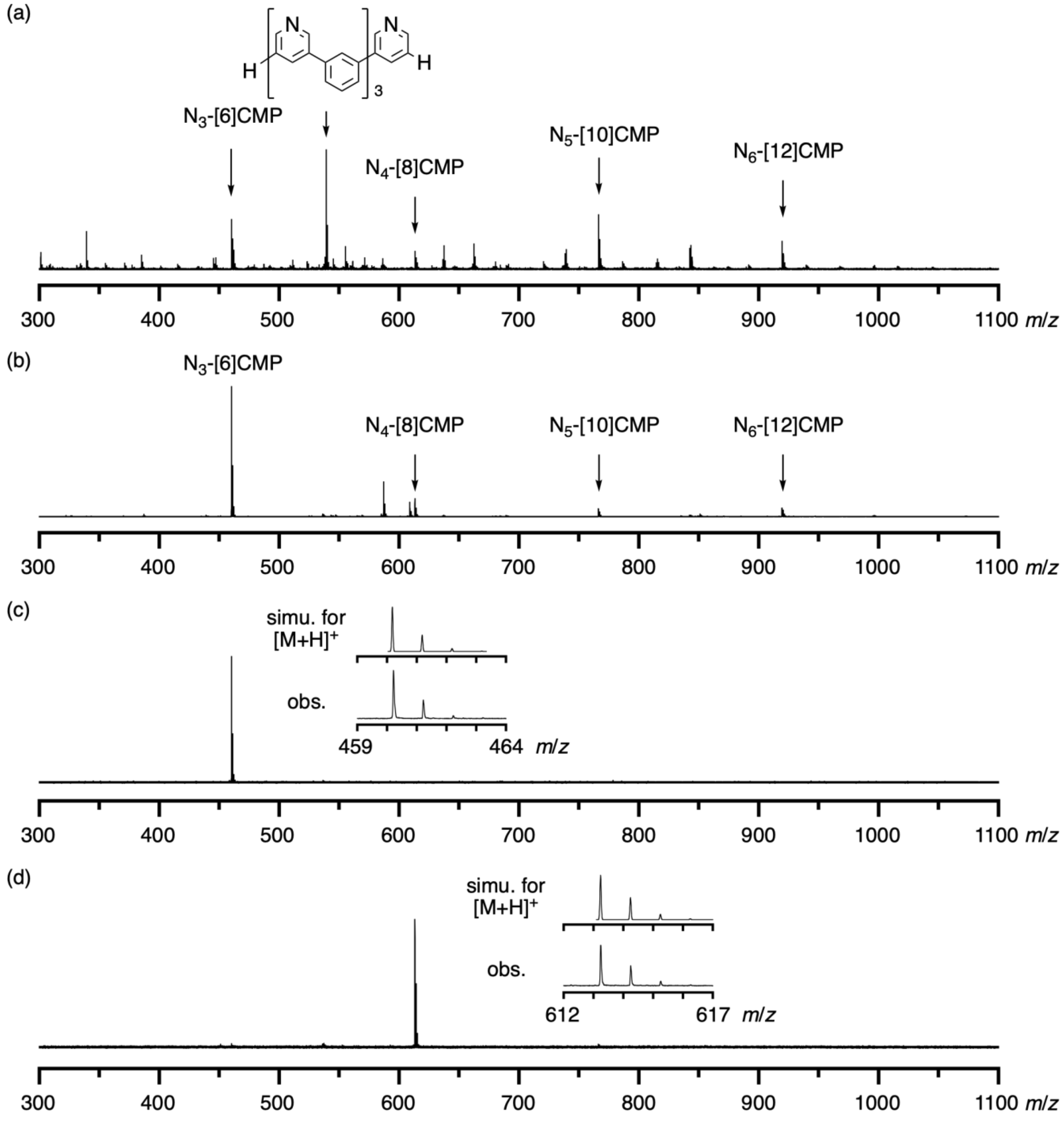

Figure S1: MALDI-TOF MS analyses (matrix: dithranol, ionization: reflector positive). Spectra of crude mixtures obtained by using (a) $Pd(PPh_3)_4$ and (b) $PdCl_2(dppf)•CH_2Cl_2$ as a catalyst. Spectra of (c) N_3-[6]CMP (**3a**) and (d) N_4-[8]CMP (**3b**) after the purification.

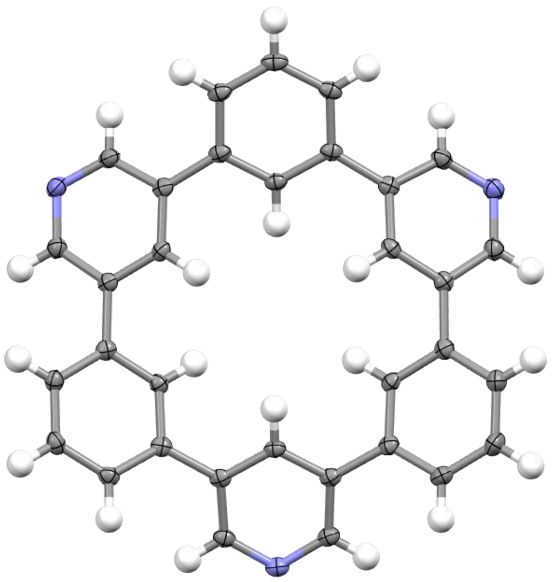

Figure S2: ORTEP diagram of a crystal structure of N_3-[6]CMP (**3a**). Thermal ellipsoids are shown at the 50% probability level.

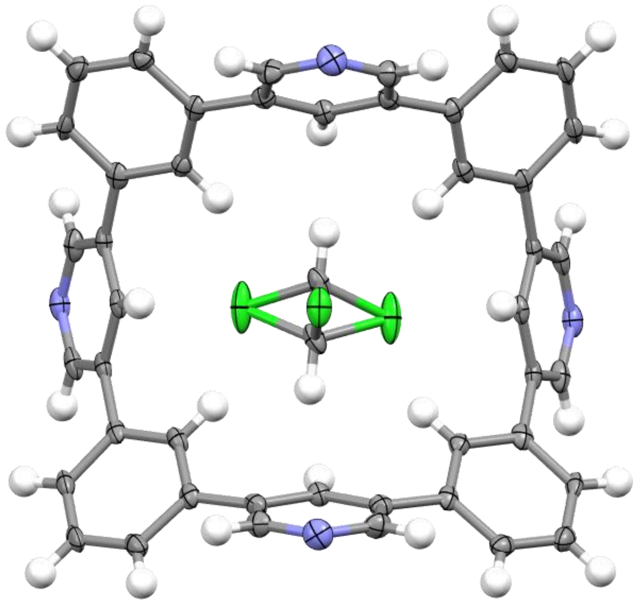

Figure S3: ORTEP diagram of a crystal structure of N_4-[8]CMP (**3b**). Thermal ellipsoids are shown at the 50% probability level.

Methods

General

Flash silica gel column chromatography was performed on silica gel 60N (spherical and neutral gel, 40–50 µm, Kanto). ^{1}H and ^{13}C NMR spectra were recorded on a JEOL RESONANCE JNM-ECA II 600 equipped with an UltraCOOL probe. Chemical shift values are given relative to an internal tetramethylsilane. Data are reported as follows: chemical shift, multiplicity, coupling constant in Hz and the relative integration value. High-resolution mass spectra were performed on a Bruker Daltonics autoflex speed using matrix assisted laser desorption ionization (MALDI) method with dithranol as a matrix (ionization mode: reflector positive) or on a Bruker micrOTOF II spectrometer equipped with an APCI probe equipped with a DirectProbe (DIP). UV–vis spectra were recorded at 25 °C on a JASCO V670 spectrometer.

Materials

Anhydrous DMF was purified by a solvent purification system (GlassContour) equipped with columns of activated alumina and supported copper catalyst (Q-5) [1]. All other chemicals were of reagent grade and used without any further purification. Diborylbenzene **5** was prepared by the reported procedure [2].

Crystallography

Single crystals of N_3-[6]CMP (**3a**) and N_4-[8]CMP (**3b**) suitable for X-ray crystallographic analysis were obtained by slowly diffusing hexane vapor into a $CHCl_3$/MeOH (10:1) solution of **3a** and **3b** at 25 °C. A single crystal was mounted on a thin polymer tip with cryoprotectant oil and was subjected to the X-ray diffraction studies as follows. The X-ray diffraction experiment was performed on a Rigaku XtaLAB P200 diffractometer equipped with a PILATUS200K area detector using multi-layer mirror monochromated Cu Kα radiation.

IAM refinements by SHELX

The crystal structures of **3a** and **3b** were first solved by a conventional method adopting spherical, independent atom models (IAM) [3]. The initial structures were solved by the direct method with the SHELXT program [4] and refined by full-matrix least-squares on F^2 using the SHELXL program suite [5]. The non-hydrogen atoms were analyzed anisotropically, and hydrogen atoms were located at the calculated positions and refined with a riding model. The refinement data are summarized in Tables S1 and S2.

Table S1: Crystal data of N_3-[6]CMP (**3a**) with the IAM refinements by SHELX

CCDC	2335441	
Empirical formula	$C_{33}H_{21}N_3$	
Formula weight	459.53	
Temperature	93(2) K	
Wavelength	1.54184 Å	
Crystal system	Triclinic	
Space group	*P*–1	
Unit cell dimensions	*a* = 5.8332(12) Å	α = 79.684(13)°
	b = 9.0678(16) Å	β = 89.420(16)°
	c = 21.492(4) Å	γ = 83.71(2)°
Volume	1111.6(4) Å^3	
Z	2	
Density (calculated)	1.373 mg/m^3	
Absorption coefficient	0.630 mm^{-1}	
F(000)	480	
Crystal size	0.10 × 0.04 × 0.03 mm^3	
Theta range for data collection	2.090° to 68.218°	
Index ranges	–7<=*h*<=6, –10<=*k*<=10, –25<=*l*<=25	
Reflections collected	14564	
Independent reflections	3942 [*R*(int) = 0.0294]	
Absorption correction	multi-scan	
Refinement method	Full-matrix least-squares on F^2	
Data / restraints / parameters	3942 / 0 / 325	
Goodness-of-fit on F^2	1.053	
Final *R* indices [*I* > 2sigma(*I*)]	R_1 = 0.0386, wR_2 = 0.1066	
R indices (all data)	R_1 = 0.0487, wR_2 = 0.1169	

Table S2. Crystal data of N_4-[8]CMP (**3b**) with the IAM refinements by SHELX

CCDC	2335442
Empirical formula	$C_{44}H_{28}N_4$•$CHCl_3$
Formula weight	732.07
Temperature	93(2) K
Wavelength	1.54184 Å

Crystal system	Monoclinic	
Space group	*C*2/*c*	
Unit cell dimensions	a = 23.783(15) Å	α = 90°
	b = 5.771(3) Å	β = 108.62(2)°
	c = 26.322(15) Å	γ = 90°
Volume	3424(3) Å^3	
Z	4	
Density (calculated)	1.420 mg/m^3	
Absorption coefficient	2.743 mm^{-1}	
F(000)	1512	
Crystal size	0.18 × 0.03 × 0.02 mm^3	
Theta range for data collection	3.923° to 70.629°	
Index ranges	–28<=h<=26, –7<=k<=6, –32<=l<=32	
Reflections collected	14509	
Independent reflections	3202 [R(int) = 0.0530]	
Absorption correction	multi-scan	
Refinement method	Full-matrix least-squares on F^2	
Data / restraints / parameters	3202 / 0 / 243	
Goodness-of-fit on F^2	1.082	
Final R indices [I > 2sigma(I)]	R_1 = 0.0805, wR_2 = 0.1999	
R indices (all data)	R_1 = 0.0932, wR_2 = 0.2071	

X-ray charge density analysis by XD-2016

For elucidating the difference of the electronic properties by nitrogen doping, crystallographic data of N_3-[6]CMP (**3a**) and [6]CMP (CCDC 1015816) [6] were subjected to X-ray charge density analyses. The SHELXL-refined structures and the HKL data were transferred to the XD-2016 program [7]. The structures were first converged by the IAM refinement with the XDLSM module in XD-2016. The resultant *R* factors were as follows:

$R(F)$ = 0.0382 and $Rw(F)$ = 0.0573 for **3a**;

$R(F)$ = 0.0361 and $Rw(F)$ = 0.0460 for [6]CMP.

Then, the data were refined by the aspherical model by adopting the transferable aspherical atom model (TAAM) [8] with the parameters of the University at Buffalo Pseudoatom Databank (UBDB) [9,10]. The significant improvement of the refinement was found with the deformation map ($F_1 – F_2$) and *R* factors:

$R(F)$ = 0.0269 and $Rw(F)$ = 0.0430 for **3a**;

$R(F)$ = 0.0237 and $Rw(F)$ = 0.0312 for [6]CMP.

The refinement data of X-ray charge density analyses of **3a** and [6]CMP are summarized in Tables S3 and S4.

Table S3: Crystal data of N_3-[6]CMP (**3a**) with the TAAM/UBDB refinements by XD-2016

CCDC	2335443	
Empirical formula	$C_{33}H_{21}N_3$	
Formula weight	459.53	
Temperature	93(2) K	
Wavelength	1.54184 Å	
Crystal system	Triclinic	
Space group	*P*–1	
Unit cell dimensions	*a* = 5.8332(12) Å	α = 79.684(13)°
	b = 9.0678(16) Å	β = 89.420(16)°
	c = 21.492(4) Å	γ = 83.71(2)°
Volume	1111.6(4) Å^3	
Z	2	
Density (calculated)	1.373 mg/m^3	
Absorption coefficient	0.630 mm^{-1}	
F(000)	480	
Crystal size	0.10 × 0.04 × 0.03 mm^3	
Theta range for data collection	2.090° to 68.218°	

Index ranges	–7<=*h*<=6, –10<=*k*<=10, –25<=*l*<=25
Reflections collected	14568
Independent reflections	3945 [*R*(int) = 0.0294]
Absorption correction	multi-scan
Refinement method	Full-matrix least-squares on *F*
Data / restraints / parameters	3228 / 0 / 346
Goodness-of-fit	2.24
Final *R* indices [*I* > 2sigma(*I*)]	*R*(*F*) = 0.0269, *Rw*(*F*) = 0.0430
R indices (all data)	$R(F^2)$ = 0.0391, $Rw(F^2)$ = 0.1138

Table S4: Crystal data of [6]CMP with the TAAM/UBDB refinements by XD-2016

CCDC	2335444	
Empirical formula	$C_{36}H_{24}$	
Formula weight	465.55	
Temperature	90(2) K	
Wavelength	1.54178 Å	
Crystal system	Monoclinic	
Space group	$P2_1/c$	
Unit cell dimensions	*a* = 12.289(3) Å	α = 90°
	b = 15.382(3) Å	β = 99.35(3)°
	c = 6.0239(12) Å	γ = 90°
Volume	1123.6(4) $Å^3$	
Z	2	
Density (calculated)	1.394 mg/m^3	
Absorption coefficient	0.579 mm^{-1}	
F(000)	480	
Crystal size	0.20 × 0.05 × 0.05 mm^3	
Theta range for data collection	3.65° to 67.33°	
Index ranges	–12<=*h*<=14, –18<=*k*<=18, –7<=*l*<=7	
Reflections collected	8005	
Independent reflections	1755 [*R*(int) = 0.0371]	
Absorption correction	none	
Refinement method	Full-matrix least-squares on *F*	
Data / restraints / parameters	1755 / 0 / 175	
Goodness-of-fit	1.485	

Final R indices [I > 2sigma(I)]	$R(F)$ = 0.0237, $Rw(F)$ = 0.0312
R indices (all data)	$R(F^2)$ = 0.0417, $Rw(F^2)$ = 0.0610

Visualization and analyses

Molecular structures were visualized and analyzed by Mercury CSD 4.1.0 [11] and UCSF Chimera (ver. 1.13.1) [12]. The electron densities after the TAAM/XD2016 refinements were visualized by MoleCoolQt [13]. The contour map of the deformation density was obtained by the XDGRAPH module in the XD2016 package [7], and the ESP maps were generated by MolIso [14]. All the crystallographic data were deposited in the Cambridge Crystallographic Data Centre (CCDC 2335441-2335443). The data can be obtained free of charge from The Cambridge Crystallographic Data Centre via www.ccdc.cam.ac.uk/data_request/cif.

Spectra

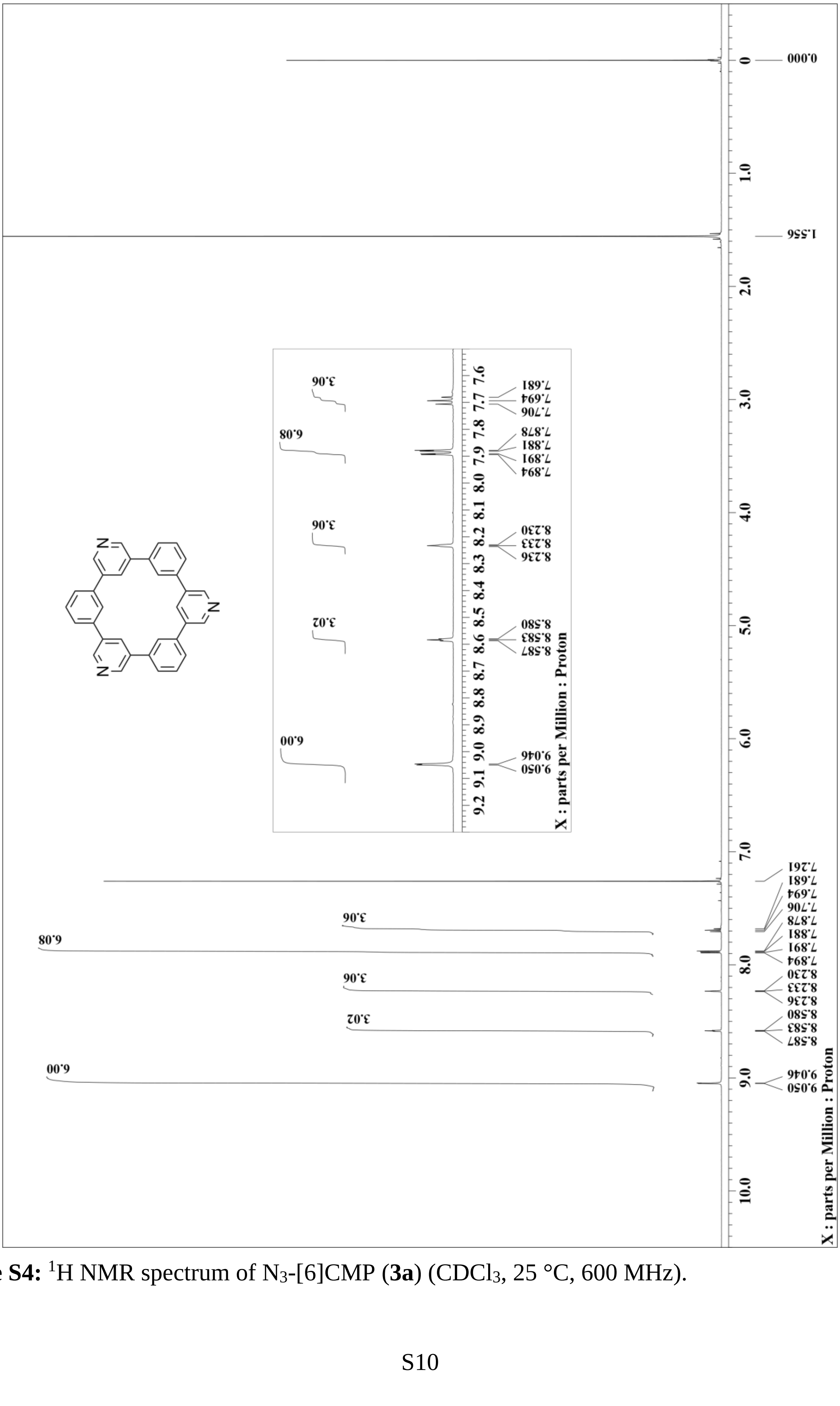

Figure S4: ^{1}H NMR spectrum of N_3-[6]CMP (**3a**) ($CDCl_3$, 25 °C, 600 MHz).

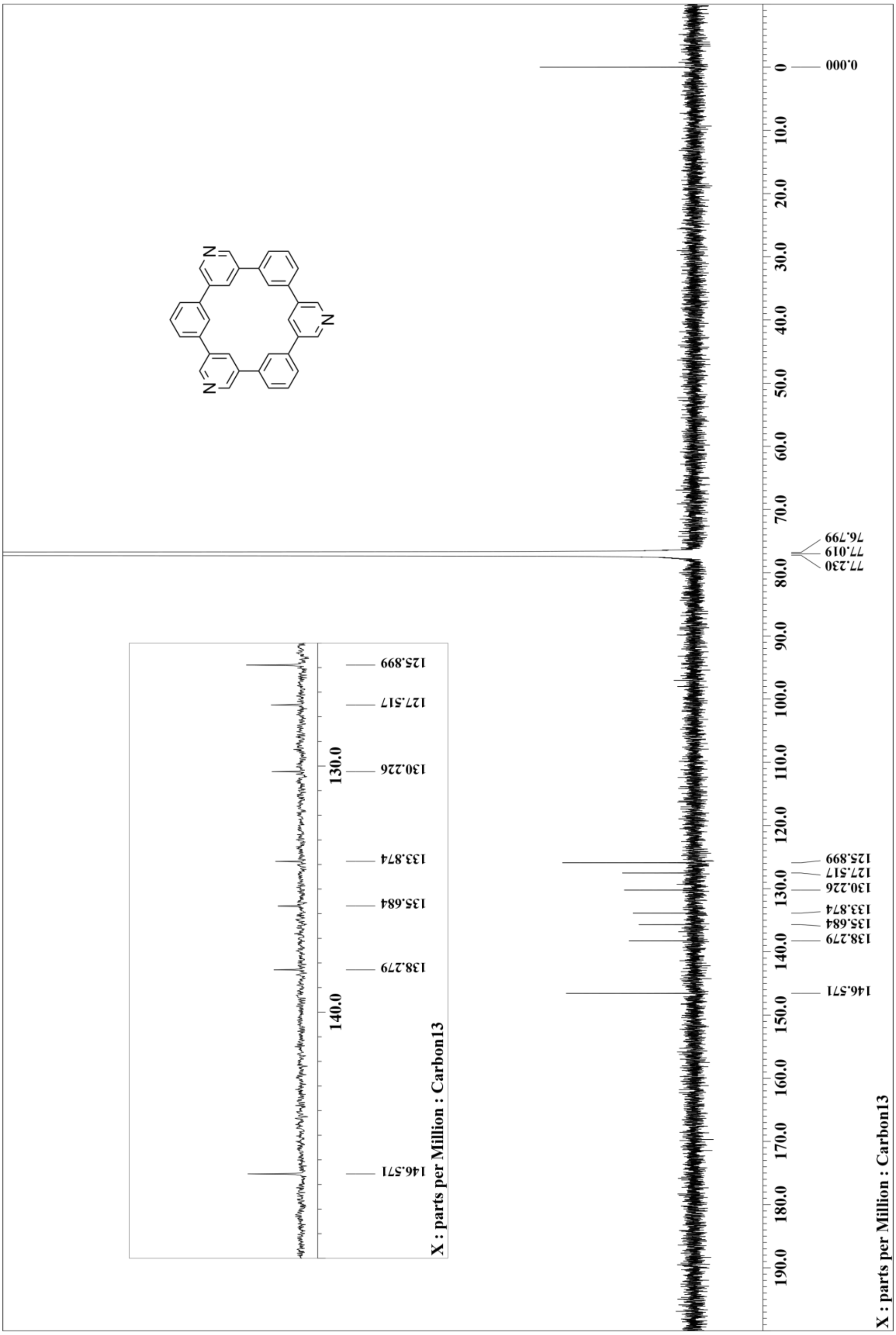

Figure S5: ^{13}C NMR spectrum of N_3-[6]CMP (**3a**) ($CDCl_3$, 25 °C, 151 MHz).

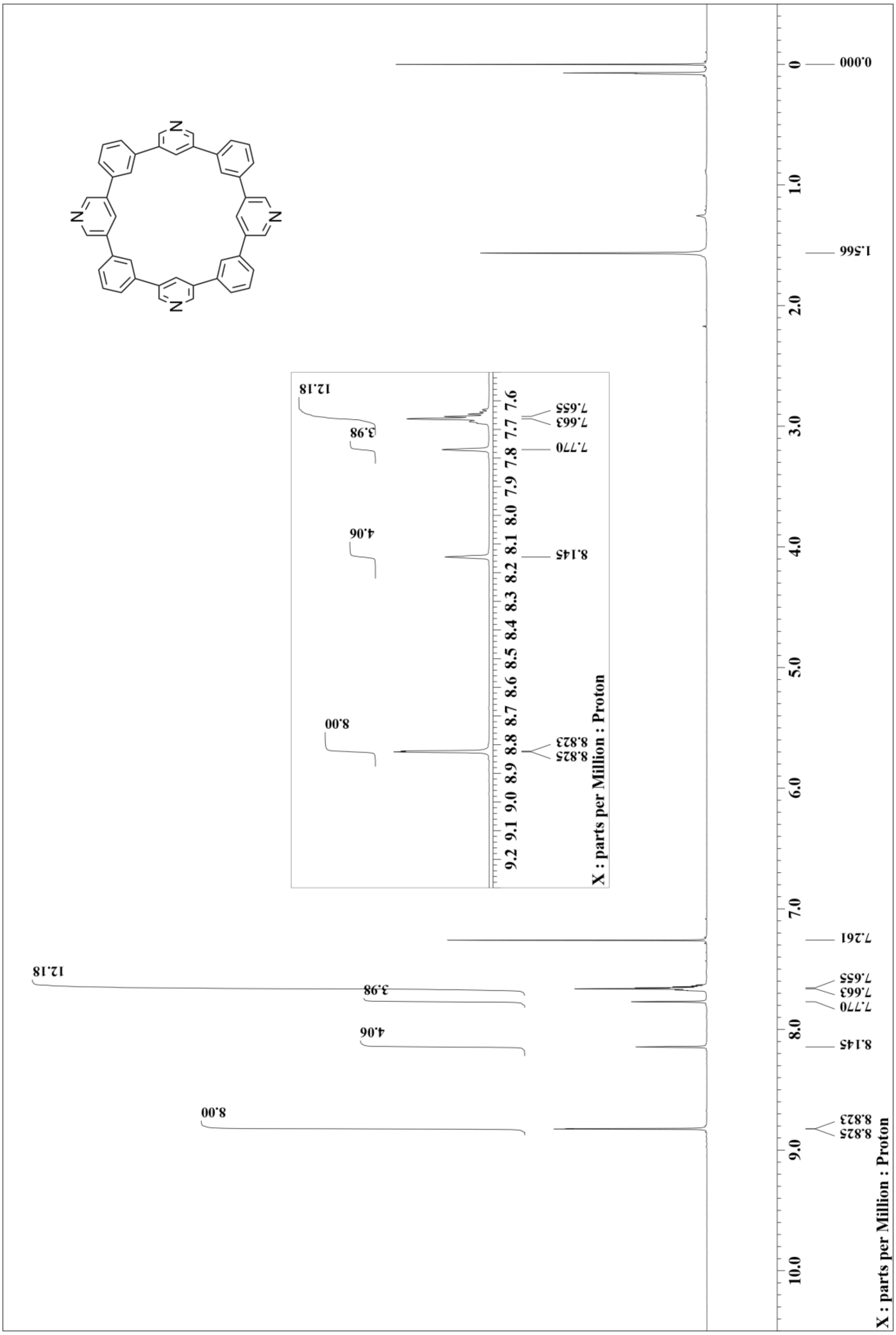

Figure S6: ^{1}H NMR spectrum of N_4-[8]CMP (**3b**) ($CDCl_3$, 25 °C, 600 MHz).

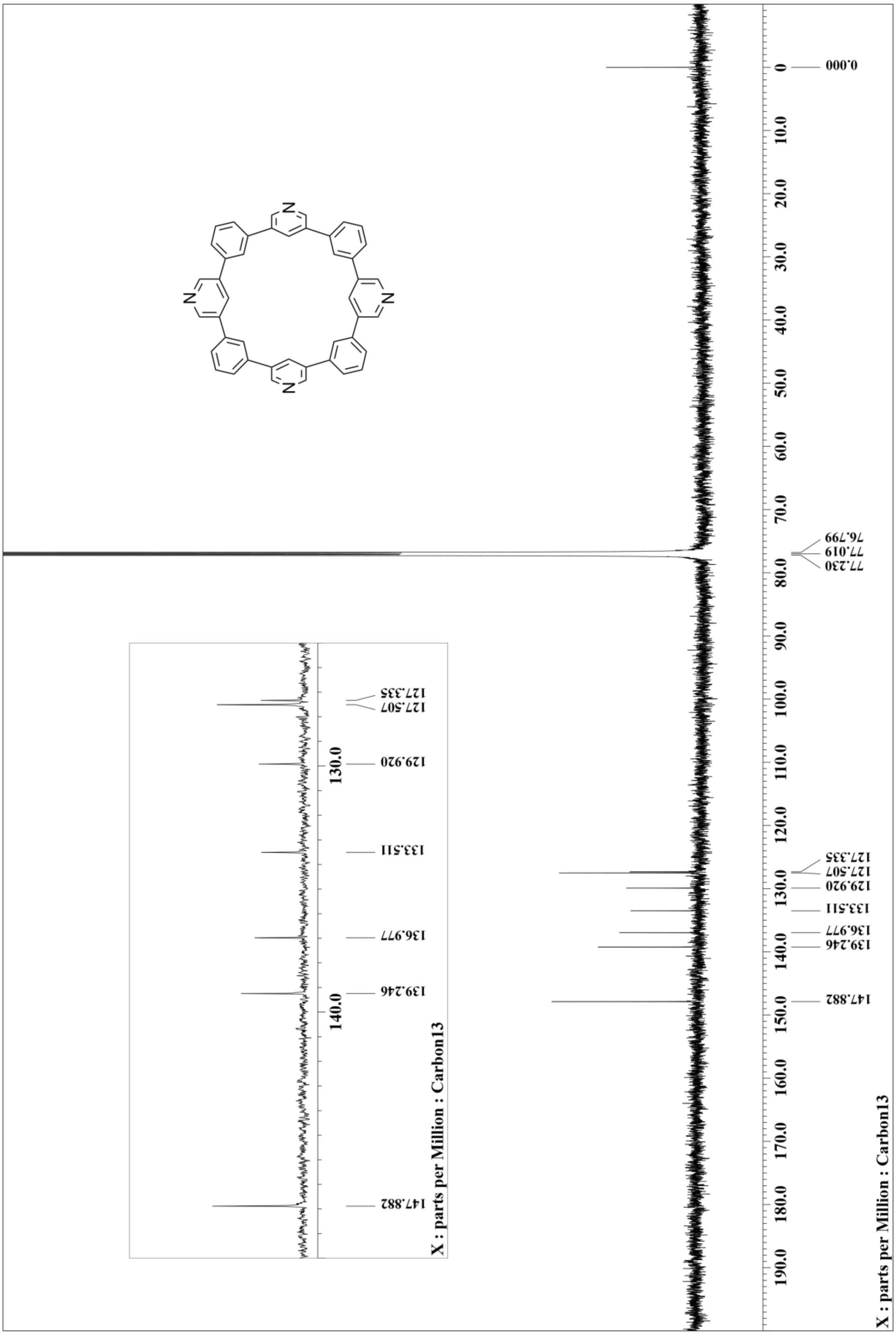

Figure S7: ^{13}C NMR spectrum of N_4-[8]CMP (**3b**) ($CDCl_3$, 25 °C, 151 MHz).

Supplementary references

1. Pangborn, A. B.; Giardello, M. A.; Grubbs, R. H.; Rosen, R. K.; Timmers, F. J. *Organometallics* **1996**, *15*, 1518-1520.
2. Sun, Z.; Ikemoto, K.; Fukunaga, T. M.; Koretsune, T.; Arita, R.; Sato, S.; Isobe, H. *Science* **2019**, *363*, 151-155.
3. Doyle, P. A.; Turner, P. S. *Acta Crystallogr. A* **1968**, *24*, 390-397.
4. Sheldrick, G. M. *Acta Crystallogr. A* **2015**, *71*, 3-8.
5. Sheldrick, G. M. *Acta Crystallogr. C* **2015**, *71*, 3-8.
6. Xue, J. Y.; Ikemoto, K.; Takahashi, N.; Izumi, T.; Taka, H.; Kita, H.; Sato, S.; Isobe, H. *J. Org. Chem.* **2014**, *79*, 9735-9739.
7. Volkov, A.; Macchi, P.; Farrugia, L. J.; Gatti, C.; Mallinson, P.; Rihter, T.; Koritsánszky, T. *XD2016, a computer program package for multipole refinement, topological analysis of charge densities and evaluation of intermolecular energies from experimental and theoretical structure factors*; University at Buffalo; State University of New York; University of Milano; University of Glasgow; CNR-ISTM; Middle Tennessee State University, 2016.
8. Brock, C. P.; Dunitz, J. D.; Hirshfeld, F. L. *Acta Crystallogr. B* **1991**, *47*, 789-797.
9. Koritsanszky, T.; Volkov, A.; Coppens, P. *Acta Crystallogr. A* **2002**, *58*, 464-472.
10. Dominiak, P. M.; Volkov, A.; Li, X.; Messerschmidt, M.; Coppens, P. *J. Chem. Theory Comput.* **2007**, *3*, 232-247.
11. Macrae, C. F.; Bruno, I. J.; Chisholm, J. A.; Edgington, P. R.; McCabe, P.; Pidcock, E.; Rodriguez-Monge, L.; Taylor, R.; van de Streek, J.; Wood, P. A. *J. Appl. Cryst.* **2008**, *41*, 466-470.
12. Pettersen, E. F.; Goddard, T. D.; Huang, C. C.; Couch, G. S.; Greenblatt, D. M.; Meng, E. C.; Ferrin, T. E. *J. Comput. Chem.* **2004**, *13*, 1605-1612.
13. Hübschle, C. B.; Dittrich, B. *J. Appl. Cryst.* **2011**, *44*, 238-240.
14. Hübschle, C. B.; Luger, P. *J. Appl. Cryst.* **2006**, *39*, 901-904.
